# Supplementary material for: Predicting visual function by interpreting a neuronal wiring diagram
Source: Nature. 2024 Oct 2;634(8032):113–23. doi: 10.1038/s41586-024-07953-5 (PMC11446822; doi:10.1038/s41586-024-07953-5)
Supplement: Supplementary file 6 — CRF and ERF predictions for individual TmY4 and TmY9 cells. Analogous to Supplementary Data 3, but for TmY target types. Shown are the top four monosynaptic pathways, the strongest pathway passing through each of the top ten intermediary types (ranking from Extended Data Fig. 7), and the trisynaptic pathway Tm1–TmY–Dm3–TmY (see the section entitled Prediction of spatial normalization). [file 41586_2024_7953_MOESM6_ESM.zip › DataS4/TmY9q/720575940609809476.pdf]

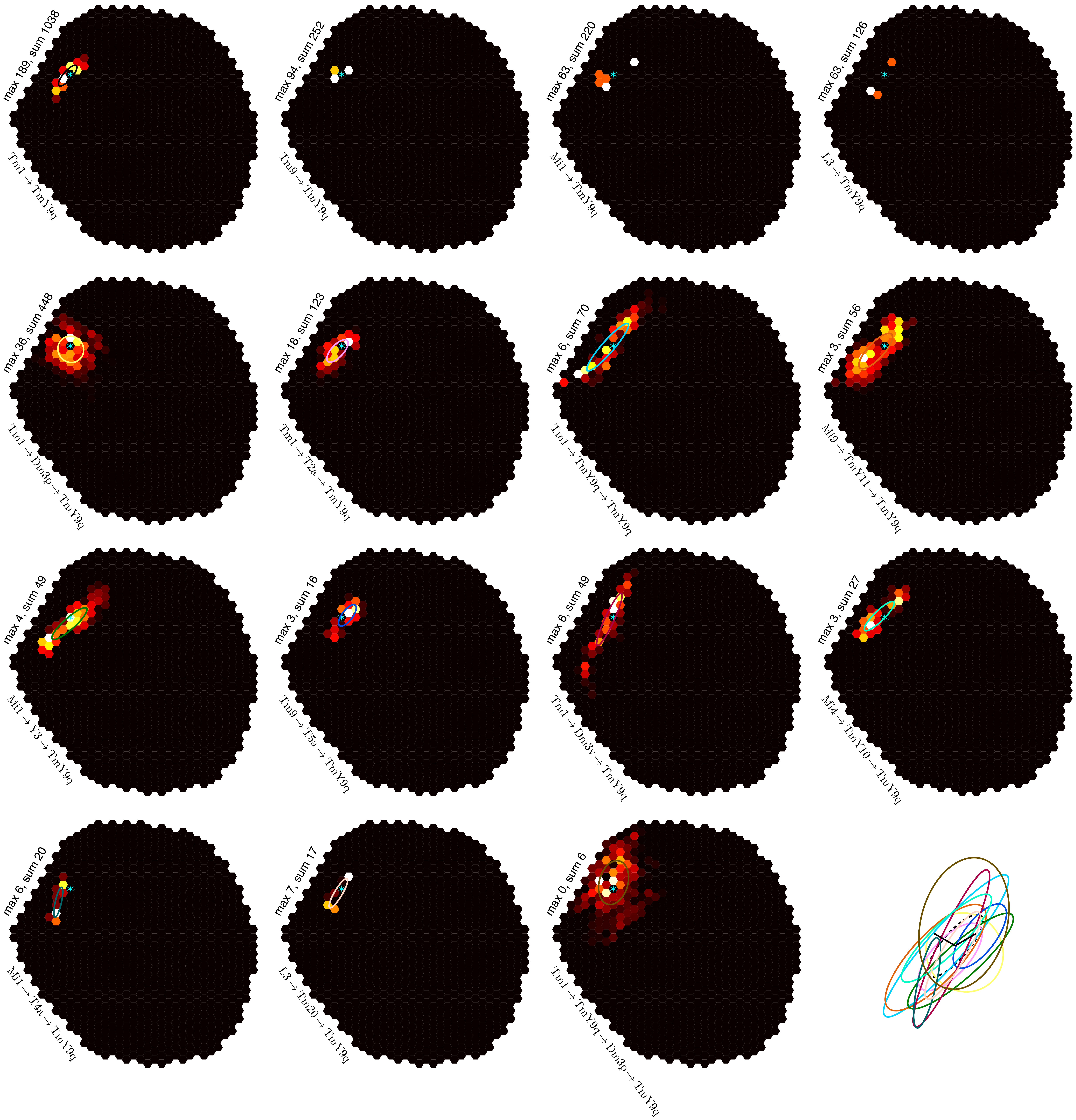
max 189, sum 1038
Tm1 → TmY9q
max 94, sum 252
Tm9 → TmY9q
max 63, sum 220
Mi1 → TmY9q
max 63, sum 126
L3 → TmY9q
max 36, sum 448
Tm1 → Dm3p → TmY9q
max 18, sum 123
Tm1 → T2a → TmY9q
max 6, sum 70
Tm1 → TmY9q → TmY9q
max 3, sum 56
Mi9 → TmY11 → TmY9q
max 4, sum 49
Mi1 → Y3 → TmY9q
max 3, sum 16
Tm9 → T5a → TmY9q
max 6, sum 49
Tm1 → Dm3v → TmY9q
max 3, sum 27
Mi4 → TmY10 → TmY9q
max 6, sum 20
Mi1 → T4a → TmY9q
max 7, sum 17
L3 → Tm20 → TmY9q
max 0, sum 6
Tm1 → TmY9q → Dm3p → TmY9q
